# Primary care in rural areas: a qualitative study on medical students’ images and experiences of working in rural areas in southern Germany

**DOI:** 10.1186/s12875-024-02677-x

**Published:** 2024-12-16

**Authors:** Jan Gehrmann, Niklas Barth, Tom Brandhuber, Pascal O. Berberat, Sophie Gigou, Antonius Schneider

**Affiliations:** 1 Institute of General Practice and Health Services Research, TUM School of Medicine and Health, Department Clinical Medicine, Technical University of Munich, Munich, Germany; 2 Chair of Social Determinants of Health, TUM School of Medicine and Health, Department Health and Sport Sciences, Technical University of Munich, Munich, Germany; 3 Department of Sociology, Faculty of Social Science, Ludwig-Maximilians-University Munich, Munich, Germany; 4 Medical Education Center, TUM School of Medicine and Health, Department Clinical Medicine, Technical University of Munich, Munich, Germany

**Keywords:** Primary care, General practitioner, Medical education, Professionalisation, Qualitative methods, Health services research, Sociology, Rural areas, Spatial methods

## Abstract

**Background:**

Rural areas are increasingly moving back into the focus of social research, especially in the context of health care. As the shortage of general practitioners (GP) in rural areas is a significant challenge in Germany, there are several programs to counteract underuse effectively, acutely, and sustainably. One of those programs is ’Beste Landpartie Allgemeinmedizin’ (BeLA), which was developed to strengthen primary care in rural areas and to sustainably promote young doctors to work as general practitioners in rural regions through didactical and financial support. The program includes an accompanying qualitative study exploring the motivational structures of medical students from a sociological perspective. For this study, the nexus of working in rural areas from the perspective of medical students with different forms of rural experiences was of interest.

**Methods:**

Qualitative interviews have been conducted at regular intervals on an ongoing basis since 2020 to investigate motivational retention effects during the program. The current 33 interviews were analysed using the sociological conceptual framework of spatial methods.

**Results:**

The images and experiences of working in rural areas condition medical education in various ways. In addition to general images of living and working in rural areas in a biographical dimension, the idea of working as a GP in rural areas includes images of specific medical competencies and is conditioned by different medical tasks. From such a perspective, the images and attributions of working in primary care in a rural region demonstrate particularities, challenges, and the potential attractiveness of working in rural regions.

**Discussion:**

The images and experiences of rural areas condition medical education in various ways and shape the expectations and the decision-making of possibly working in rural areas. The particularities, opportunities, and challenges of working in rural areas, which relate to both professional aspects and social life, are a major factor in the attractiveness of a potential rural practice. Didactical and educational curricula need to adapt the various attributions of working in rural areas.

**Supplementary Information:**

The online version contains supplementary material available at 10.1186/s12875-024-02677-x.

## Background

Rural areas are increasingly moving back into the focus of social science research [1]. The nexus of rural areas is particularly evident in the context of health care [2, 3]. The discussion ranges from the analysis of structural conditions and disparities to health policy issues and concrete measures and programs to counteract the underuse of health care services or the shortage of health care professionals effectively, acutely and sustainably. In the field of global health, rural primary care and generalist medicine have emerged as a critical area of focus, particularly in regions where access to specialised healthcare is limited. Rural generalist practitioners are healthcare providers who offer a broad spectrum of services to meet the diverse needs of rural populations. Studies like Schubert et al.‘s scoping review play a vital role in understanding how different countries approach the education, training, and deployment of generalists to address healthcare disparities in rural areas [4]. Such work is crucial as it informs policy decisions and helps shape training programs to address rural healthcare delivery’s unique challenges. Other studies complementing the review emphasise the effectiveness of contextually adapted educational programs and the critical role of continuous professional development for rural practitioners, collectively underscoring the complexity of delivering high-quality healthcare in rural settings and the need for ongoing research to optimise rural generalist education and support systems globally [5–8]. Various national and international studies have already addressed why people choose to specialise in general practice, and it is shown that a majority are students from rural regions who aim for specialisation in general practice and a practice in the countryside [9–12]. Furthermore, a connection can be recognised between the place of birth, the place of study and the GP activity [13]. Overall, the shortage of GPs in rural areas is a significant challenge in Germany [9].

The context of this study is the German program ‘‘Beste Landpartie Allgemeinmedizin’’ (BeLA), funded by the Bavarian State Ministry of Health and Care. The program aims to strengthen rural primary care and to sustainably promote young doctors to work as a GP in rural areas [14–16]. The program is aligned with international programs. Programs for the education and training of (future) GPs in rural areas have been implemented internationally, particularly in North America and Australia [12, 17–22]. The BeLA program aims to bind students to rural regions through didactic and financial support at an early stage of their training to encourage them to work as GPs in the region at a later stage [14]. The regions covered by the program are Eichstätt/ Kösching, Mühldorf am Inn, and Dillingen in southern Bavaria in Germany. In all regions there are smale to large towns, medium-sized hospitals and healthcare services with several GP and specialists practices, all of which are already or potentially statistically affected by underuse. As part of the program, all participants complete part of their education in the region’s clinic and GP practices in the region. In previous studies, we were able to highlight the complexity of motivations regarding the possibility of later working as a GP in that region and the retention effects of a program such as BeLA [15]. Thus, over 85% of the participants in the program remain for their further training in general practice. The details of the inpatient training and the core didactic elements of the program have already been presented earlier [14]. The program includes a qualitative study exploring the motivational structures of medical students from a sociological perspective.

There have been several studies, mainly with a social research focus, analysing the perceptions of rurality and the preferences regarding rural teaching and clerkship formats among medical students. Other studies have shown that medical students need to experience working in primary care to increase the attractiveness of choosing general medicine [15, 23, 24]. As van der Zwet et al. [23] point out, medical education needs to adapt to the *socio-cultural* context for developing a professional identity allowing medical students to *experience* professional medical work. There have also been studies, mainly from a social research background, that focus on professionalisation and the biographic dimension of retention effects [25]. In general, there are only a few studies combining those perspectives. Armstrong pointed out that the spatial and temporal features of GPs’ perceptions of their work are integrally bound up with the social relations and activities of that work [26]. Another aspect is the different assessments of GPs as to whether they work in urban or rural areas [27].

Additionally, studies have shown regional differences in the patient population for primary care differ between urban and rural areas [28–30]. In social research, there is a new interest in spatial methods, especially in health care research focusing on regional differences and disparities and the differences between urban and rural areas from a national and international perspective. Spatial methods are an umbrella term for various methods and methodologies, with quantitative and qualitative methods approaching space as the object of research [31, 32].

In this study, we connected the research interest regarding the motives of medical students in the BeLA program with the perspective of the sociological conceptual framework of spatial methods. Therefore, rurality as a specific configuration of space is a lens through which to address sociological research questions [31]. In this study, we focussed on the narrative imagining of rurality as well as the experience of rurality of the participants. This study aims to present the images and experiences of rurality of medical students during medical education and how this influences their conceptions of medical work in general. The research question is as follows: How do medical students who are potentially willing to work in primary care in rural regions frame rurality?

## Methods

### Study design

In this qualitative study, semi-structured narrative interviews have been conducted with participants at regular intervals since 2020 at different times in the program to investigate intraspecific motivational commitment effects during the program. For this study, we analysed data on a cross-sectional level, as we aimed to identify different narratives in an exploratory way [33]. We analysed the narrative patterns in the material by adapting the conceptual framework of spatial methods (see Data Analysis).

This study is reported following the Consolidated Criteria for Reporting qualitative Research (COREQ) (Supplement 1) [34]. The quotes included have been translated into English (JG, AS, SG) with due diligence and are cited with an indication of a pseudonymised participant number.

### Recruiting and sampling

To be eligible for participation in the qualitative study, students needed to be enrolled in the BeLA Program. The participants’ data were available when they became part of the program. Participants needed to be at the end of their medical studies (at least 5th semester or alternatively in the PJ) to take part in the BeLA program. The medical studies are followed by the practical training year (German: Praktisches Jahr (PJ)) and the specialisation (German: Facharzweiterbildung (WB)). The specialisation choice occurs after medical school following the PJ [14]. Recruitment was carried out by two researchers (JG, NB) and participants were contacted via email or phone. There was no additional relationship between participants and researchers prior to the study. However, the study design and the integration of participants in the network of the Institute through the program created acquaintances. The study aimed at a full survey of all participants. Written information about the project, including the researchers involved, was mailed to potential participants. All participants gave written consent. The date and time of the telephone interview were confirmed individually (JG, NB). No reimbursement was given as students have already been remunerated in the BeLA program.

### Data collection

Semi-structured guide-based narrative interviews were conducted via telephone by two researchers from the study team (JG, NB). All interviewers (two males) had a background in sociology, were experienced interviewers, and conducted the interviews either from the Institute or their home office. The participants were interviewed at their workplace or at home. The interview topics based on the research question, existing research, and preliminary research. NB and AS created the interview guides. The interview guides can be found in the Supplement (Supplement 2). The guides contained an open narrative stimulus. This allowed the participants to choose how to begin and organise the interview [35, 36]. The opening was followed by further topic-related questions on the evaluation of the project and expectations and experiences within the project, including practical medical work.

All interviews were digitally recorded and transcribed verbatim. No field notes were made. All data collected were pseudonymised, digitally saved, and stored on secure servers at the Institute of General Practice and Health Services Research at TU Munich.

### Data analysis

In total, 33 interviews were analysed for this study. We analysed data on a cross-sectional level, aiming to identify different narratives in the data in an exploratory way [33]. The conceptual framework of spatial methods served as a conceptual guidance inspiring data analysis illuminating the different descriptions, images and configurations of rurality in the participants’ narratives [32, 37]. The spatial methods perspective following Baur et al. integrates qualitative, quantitative, and also cartographic approaches to analyse spatial phenomena. Spatial methods focus on the importance of space and place in social processes, emphasising interdisciplinary integration and the consideration of different spatial scales and figurations. The approach links theoretical concepts with empirical research and methodological practices to achieve a more comprehensive understanding of the role of space and place in social research [32]. In this study, we were interested in the narrative semantics and descriptions of rurality and the associated attributions to primary care practice in rural areas. We chose purposeful cases and quotes of the material to present in the results.

Before data analysis, we became familiar with the interviews via the transcripts. The coding of the material is based on the initial and focused coding, according to Charmaz [38]. The initial coding served as data familiarisation and, thus, a first exploration of the material. This step aimed at identifying the narratives patterns, circumstances, and history of the individual participant as well as how and regarding which aspects of rurality are mentioned by the participants in the interview. The first initial codes were developed in this step, being aware that they were emergent and could be changed. The initial coding served as an exploration, also following the conceptual framework of spatial methods. A final set of codes and categories was created in the focused coding. The focused coding then led to the codes’ selection, initial categorisation and weighting of the codes. Individual passages from the interviews were selected and combined into further categories in order to compare and contrast. The categories were rechecked by comparing them again to the material, the context and the passage of the coded segment within the interview. The analysis was conducted and reflected upon within the research team (JG, NB, SG, and AS). All data were analysed until the consistency of findings enabled thematic saturation. The material was analysed using MAXQDA 2020.

The systematic analysis of this study focused on the narratives of the participants regarding the images and experiences of rural areas. The analysis did not focus on the individual narrative cases of the interviewees. It aimed to structure the participants’ narratives to provide a cross-section of their images of rurality. Due to the mediality of the interviews [39], the methodological gaze did not set out in search of latent structures of meaning that could then be retrieved from the depths of the text. The analysis was directed towards the communicative ‘‘selection frames’’ [40]. The fact that specific narrative patterns occurred in the material – and others did not – was already crucial information [39]. Following Baur et al. [29], we focussed on the narrative imagining and semantics of rurality, the experience of rurality in one’s biography and the representation of rurality in the participants’ images of medical work in rural areas. For example, in the first step, general descriptions of rural life and work in the interviews were coded to initially structure the nexus of rurality, and in the next step, these could be harmonised with the participants’ ideas about their future work, life planning and the demands placed on their own medical work or career planning. The analysis showed that the participants’ images of rurality are related to the requirements of their medical education and future careers. Through this analytical perspective, we could be sensitive to semantics, notions, and imaginations of rurality and how the experience of rural areas shapes medical education. To clarify, we were interested in the narratives of medical students and did not intend to infer what primary care work is like in rural areas of southern Germany in general.

For the presentation of the results, concise statements from the participants were selected and are presented in the following.

## Results

Overall, 33 interviews with 22 participants of the program were conducted. In addition, four participants of the program did not take part in the study due to unknown reasons. Two additional participants voluntarily dropped out of the program and were also not included in the study. For further characteristics of the study participants, see Table 1.

**Table 1:** Sample characteristics (*n* = 22)

	Value ***n*** (%)
**Gender**	
Male	11 (50)
Female	11 (50)
**Rural background** (born or raised in rural area)	
Yes	19 (86,4)
No	3 (13.6)
**Training stage**	
Medical studies	6 (27.3)
Practical training year (PJ)	10 (45.4)
Specialisation (WB)	6 (27.3)
**Rural medical experience** (already completed parts of their education in one of the rural regions)	
Yes	16 (72.7)
No	6 (27.3)

### The image of rurality in general

In the narratives, general attributions of the ‘decelerated’ and slow life in rural areas are made. Additionally, there are also references to the infrastructural and financial conditions of rural regions, as the following quote illustrates:

*‘And I have the feeling that the country is a bit*,* shall I say*,* decelerated. (…) Another aspect is of course the financial aspect. If I wanted to live in Munich later on or in another big city with a family*,* it is just brutally expensive and then maybe I could live just in a flat somewhere. And in the countryside*,* it is quite realistic that I might be able to put up my own four walls and I’d actually like that.’ (I01)*.

The idea of life in rural regions is also accompanied by the desire for self-realisation and financial as well as creative opportunities in planning one’s life. This aspect also has an inherent identitarian motive and is associated with complexes such as family planning, projections on the future of one’s own children, and ideas of everyday life.

*‘So once*,* nature*,* we are both nature-loving and yes*,* you have just*,* you have that just closer to the front door than in any big city. Now we live in the centre of Munich*,* we have now also / enjoy that also*,* but it has just both its advantages and disadvantages. And somehow it feels that also again such a time in the countryside*,* that would be great. And especially for the children*,* I have the hope that they can simply*,* yes*,* again*,* yes*,* experience a different childhood than in the city. So I think country life is truly great for children. Because the relationship to nature can be further cultivated and can actually be conveyed to the children more easily than in the city. These are already such considerations.’ (I02)*.

These more traditional rural semantics persist in the narratives of the participants, with the countryside being imagined as a counterpart to the urban area. The idea or even one’s own experience of the everyday life of rurality influences the planning of further education and future life planning, including a medical career. The concept of living and working in rural areas appears as a motivational factor itself.

### Rurality as a condition for the status of the profession

In the narratives, different attributes of the profession of GPs and the different conditions and requirements for medical practice in rural regions can be analysed. The mode of narrating is mainly based on the distinction between rural and urban areas. Such differences seem crucial for understanding one´s biographical positioning regarding possibly working as a GP in rural regions. The differential status of GP is particularly emphasised here:

*‘And I also believe that the status of the general practitioner*,* especially in rural areas*,* is great.’ (I02)*.

The different status of the GP is not only a normative ideal but can be traced back to the different needs and perceptions of patients in rural regions. The different organisation of health care and the gatekeeping and ‘distribution function’ are named as central elements of the status of the GP:

*‘So I would not do general medicine in Munich if I stayed there. (…) Yes*,* I have the feeling that in Munich*,* as a general practitioner*,* you are only there to issue sick notes. That is how I see it. So everyone goes straight to the specialist. You truly only have this distribution function.’ (I03)*.

If one highlights the gatekeeping, one finds ambivalent remarks about the consequence of the mediating function in contrast to the providing function. Thus, on the one hand, the challenge of the symbol ‘issuing sick notes’ is clearly stated. On the other hand, the central role of the GP is reflected as an opportunity for realising the ideal of good care combined with a high degree of identification with the profession.

*‘because I see here in the countryside that patients want to get well very quickly and you have to be quite skilled and informed (….)*,* because*,* yes*,* people no longer insist only on conventional medicine. (….) So you really have to know your stuff and know what you are doing*,* because as I said*,* your reputation can be destroyed very quickly by word of mouth.’ (I03)*.

In urban areas, the GP is figured as the general provider who distributes patients to specialists or only issues sick notes. In rural areas, the GP is considered a general provider who needs to offer a wide range of care adapting to overall patient needs. The role of the GP is conditioned by structural factors, as there is a need for more specialists, and by the fact that the provider function of the GP in rural areas is emphasised. The expectations regarding the role of the GP is an important motivational factor for possibly working in rural areas.

Furthermore, the images of rurality regarding cultural or traditional semantics of the rural GP offer a biographical stencil for narrating one´s own biography, which enables one to critically reflect on the own profession and distance oneself from a traditional, primarily rural professional image. This topic also refers towards the expectations directed at GPs. The traditional semantics persist partly, but overall societal change is perceived. The professional role of the GPs is decoupled from the social role.

*‘There probably still is*,* but the classic doctor*,* who then also*,* yes*,* (…) at the lunch table or in the pub or wherever*,* always remains in this role and then has to be active again immediately. I think that is no longer the case and it does not have to be.’ (I04)*.

The demarcation also takes the form of a generational difference and the (structural) transformation of medical care, which necessitates a change in the professional self-description of GPs.

*‘I think the younger ones in particular are no longer prepared to be the doctor who is on call from zero to 24 hours*,* seven days a week. (…) And it is not even necessary anymore*,* because it is simply organised in*,* I think*,* better structures in the meantime*,* where the practices also work together with outpatient clinics that are connected to the hospital. (…) I have only experienced it in such a way that (…) no one burns out completely in their role as a family doctor*,* although they always have an open ear for the patients and try to care for them in the best possible way’. (I04)*

Despite the new attributions of working as a GP, the overall topic is providing adequate care, and it also focuses on the biographical history over the life course and social circumstances of the individual patient.

### Rurality as a condition for working in primary care

The images and expectations of medical students regarding working in primary care are also framed by their own biographic experiences and during their medical education. In addition, images of specific competencies of a GP in a rural region are named.

*‘I think that in the countryside you simply build up a longer-term patient relationship somehow*,* I think (…) And I think the difference is also that you simply know many patients beforehand or know them better*,* I would say*,* than in the city. And from that point of view*,* you can perhaps also assess patients better because you have known them for longer and have a different relationship than in the city. And for the patients*,* from the patient’s point of view*,* the relationship is*,* I think*,* different*,* like when you go to a different doctor every time in the city.’ (I01)*.

In particular, the image of the holistic and long-term care of patients is characteristic of rural medical work. It is named as a potential for patient care and the doctor-patient relationship.

*‘And in the countryside*,* I can imagine that you truly accompany people for a long*,* long*,* long time and are perhaps the only doctor in their environment. And accordingly*,* it is important to put yourself in their shoes*,* in the patient’s shoes*,* and to be able to tell them apart. In other words*,* to know the people at some point. And that*,* I think*,* makes a lot of difference.’ (I01)*.

The goal is to build a long-term, trusting relationship with patients. Information about the patients can also inform and improve their treatment. This requires, above all, a fundamental attitude toward one’s work and appropriate communication and interaction with the patients. Following that image, domestic and intimate contact with patients is described, which leads to different medicalised attributions. It is precisely the overly intensive doctor-patient relationship that is ambivalently negotiated.

*‘And that is just nice when you accompany patients for years*,* when you are allowed to get an insight into the home environment. And*,* yes*,* maybe you also have to go out at night sometimes*,* but the people are just so grateful to you’ (I05)*.

The gradually increasing intimacy in working in rural areas is also critically reflected in respect to the possible necessity of home visits.

*‘Yes*,* so one point where I have always noticed that there are perhaps discrepancies between my idea of being a family doctor and how it is actually lived in my practice is the issue of home visits. (…) So you sometimes get to see things or experience patients in the role that they live in everyday life. Sometimes I thought to myself*,* I do not know if I really wanted to continue to do this to the extent that it is still done in some places.’ (I04)*.

## Discussion

In this study, we showed that rurality conditions medical education in various ways. Our results offer a perspective on the semantics, images, and experiences of medical students and how this shapes their medical education. Various national and international studies have already addressed why people choose to specialise in general practice [9–12]. Other studies have also shown that rurality is a significant factor for professionalisation, as medical students attending a rural campus or spending time in a rural area are more likely to practice in nonmetropolitan areas upon graduation [41]. In many cases, students of a rural origin are predisposed to want to work in rural areas, which could confound these positive findings. Therefore, the experience of rurality, despite being conditioned biographically through one’s heritage or the experience of rurality during medical education, correlates with possible retention. A perspective missing was the one focusing on variables that might influence rural practice decisions and what images and attributions as well as experiences the medical students have working in rural areas. A scoping review by Schubert et al. (2018) examines international approaches to rural generalist medicine, highlighting the diversity in how rural generalist roles are defined and implemented across different countries. The study concludes that successful models typically include targeted training programs, support systems, and community engagement, which enhance the recruitment and retention of rural healthcare professionals. The review underscores the importance of tailored, context-specific strategies to address the unique healthcare challenges faced in rural areas globally [4]. Our data indicate that programs like BeLA need to be implemented at the early stages of the education process to address students in a promising manner. The results may also indicate how it is possible to motivate young medical students to work in rural areas by offering experiences of working professionally in rural areas. Additionally, it seems to be of importance to create a didactical support frame enabling to experience the particularities, challenges and advantages of potentially working in rural areas.

As our results demonstrate, the (potential) attractiveness of working as a GP in rural areas is based on the requirements of working in rural areas. Rural general medicine is described as more holistic and diverse, requiring various medical competencies, which the participants perceived as attractive and challenging. Educative curricula must adapt to the various circumstances and attributions of working in rural regions [8]. The nexus of rurality (as a figuration of space) in this study touches on various aspects, starting from general images of rurality to concrete images and experiences regarding medical work and competencies. In the course of the interviews, various motives emerge regarding possible future medical work in a rural region. The participants addressed demands on the fit between professional work and private life, private life planning, and professional aspects such as the possibility of a more intensive, holistic and long patient relationship as factors in favour of possibly working in rural areas in the long-term. The importance of medical students’ diverse and complex motive structures in terms of the potential work in primary care has already been shown by other studies [8, 42–44]. We have already been able to demonstrate various ideal types of narratives regarding professionalisation [39]. It has already been pointed out in other studies that medical education is a demanding phase interwoven with various life events and involves the challenge of continuously acquiring professional competencies and developing one’s professional identity as a specialist [43]. Recognising the characteristics of the participants, we can show that the choices of medical students are interwoven with their concepts of and connection to rural areas. The distinction between primary care in rural and nonrural areas is dominant in the narratives and is also shown by other qualitative studies [27, 30]. Other studies have also emphasised that rural GPs described themselves as medical companion with an intensive doctor-patient relationship [27] or examined aspects of GPs’ duties and functions in rural areas from rural GPs’ perspective [45]. These particularities, opportunities, and challenges of working in rural areas seem to make the potential work as a rural GP attractive to the participants in our study.

Additionally, studies have shown the effects of regional differences in the patient population for primary care, complementing the results of this study regarding the different attributions and requirements of working in rural areas stated by the participants [28–30]. Hansen et al. stated that GPs could compensate for the specific needs of their patients through medical training aligned with the requirements of the region in which they work. In their mixed methods study, the study demonstrated that GPs working in rural areas in northern Germany would mainly need skills regarding the care of children or non-compliant patients, while urban GPs would mainly be confronted with treating patients with psychiatric, social and cultural problems [30].

Being led by the perspective of spatial methods in the qualitative data, we can show that space conditions medical education in various ways. In particular, the demarcation from traditional semantics of medical work can be understood as a ‘re-figuration’ [46] of the image of GPs, which is reinforced by different attributions of professionalism and generational differences. The demarcation from mostly traditional images and semantics of the rural GP in the narratives indicates a generational change and a changed professional self-image as a doctor, in which the separation of professional and private life and the issue of work-life balance are vital motives. An intriguing aspect of the findings is how traditional rural semantics persist, with the countryside being imagined as an idyllic counterpart to the urban city. Other studies have also demonstrated these different attributions [39, 47]. The contrast also influences the perceptions of medical work in rural areas and is a motivational factor to consider working in rural areas in the long term. Thus, the study results are of interest for recommendations and health policy measures for the sustainable development of primary care in rural areas. Our results also imply that the investment in medical education in rural areas should not start with the programs only but also within the first semesters as well as the admission to the university.

Furthermore, this study opened up health services research to spatial methods. With the emerging perspectives and application areas of spatial methods, this methodological approach offers a variety of great opportunities for further research. We explored the semantics of the rural in the qualitative data and combined it with the conceptual framework of spatial methods. This approach is promising as it enables the identification of motives related to the images, experiences, and attributions of working in rural areas and links them to the issue of medical education and the choice to potentially work in rural areas.

This study has several limitations. One limitation is that the participants were at different stages of their education and had different levels of experience of living and working in rural areas, as some were born and raised in rural areas, while others were not (see Table 1). So, there is a need for a more systematic analysis also in longitudinal perspectives of how these images and experiences shape professionalisation in the long term over the whole educational process. Overall, a central limitation is that our study is located within a particular region in southern Bavaria in Germany. The regions are deprived with small to large cities and large catchment areas. Comparisons within other German regions with different sociodemographic and infrastructural structures are necessary and would be beneficial to discover similarities and differences. Such an approach could also yield more generalisable results for the entire German healthcare system. Likewise, a globally orientated comparison would be necessary as the overall challenge of rural health care and promoting primary care is a challenge in many other countries.

## Conclusion

This study highlights the potential influence of medical students’ perceptions and experiences of rural areas on their education and career decisions, particularly regarding primary care practice in rural regions. The findings suggest that rurality is not merely an abstract concept but a lived experience that shapes students’ professional identities and aspirations. The decision to work in rural areas is deeply intertwined with personal and biographical factors, as well as the perceived status and role of the GP in these settings. The study underscores the need for medical curricula to incorporate rural experiences and address the unique challenges and opportunities of rural practice. By doing so, educational programs can better prepare and motivate students to pursue careers in rural health care, potentially addressing the shortage of GPs in these regions. The importance of such programs has already been shown in various countries. Future research should continue to explore the complex interplay between place, professional identity, and career choice, utilising spatial methods to deepen our understanding of these dynamics.

## Electronic supplementary material

Below is the link to the electronic supplementary material.

Supplementary Material 1

Supplementary Material 2

## Data Availability

The data generated and analysed during the current study are not publicly available due to the institution’s data protection guidelines but are available from the corresponding author upon reasonable request.

## Abbreviations

‘BeLA’: Beste Landpartie Allgemeinmedizin
‘GP’: General practitioner
‘COREQ’: Consolidated criteria for reporting qualitative research
‘PJ’: German ‘Praktisches Jahr’ (practical training year)
‘WB’: German ‘Facharztweiterbildung’ (specialisation)
