# Supplementary material for: Primary care in rural areas: a qualitative study on medical students’ images and experiences of working in rural areas in southern Germany
Source: BMC Prim Care. 2024 Dec 16;25:416. doi: 10.1186/s12875-024-02677-x (PMC11648291; doi:10.1186/s12875-024-02677-x)
Supplement: Supplementary file 1 — Supplementary Material 1 [file 12875_2024_2677_MOESM1_ESM.docx]

**COREQ Reporting Checklist**

| Domain 1: Research team and reflexivity | |
| --- | --- |
| Personal Characteristics | |
| 1. Interviewer/facilitator | Detailed in methods section “Data collection”:  Interviewer: Jan Gehrmann (JG), Dr. Niklas Barth (NB) |
| 1. Credentials | JG: Master of Arts Sociology  NB: PhD in Sociology  Tom Brandhuber (TB): Physician/ General Practitioner  Pascal Berberat (PB): Dr. med./ Prof.  Sophie Gigou (SG): Master of Arts Sociology  Antonius Schneider (AS): Dr. med./ Prof. |
| 1. Occupation | JG, NB, SG, TB: research associates  PB: Professor (Medical Education)  AS: General Practitioner, Professor (General Practice and Health Services Research), project coordinator |
| 1. Gender | JG, NB, TB, PB, AS: Male  SG: Female |
| 1. Experience and training | Interviewers had significant experience in qualitative research and were led by an experienced researcher in health services research (AS). Additionally, they attended external workshops and informal trainings at the Institute.  Experience:  JG: University degree in Sociology; significant experience in qualitative research and interviews, realized qualitative interviews  NB: University degree and PhD in sociology; significant experience in qualitative research and interviews, realized qualitative interviews  TB: physician, experience in medical education and health services research  PB: Professor of Medical Education; conceptualized and realized several studies  SG: University degree in sociology; some experience in qualitative research  AS: conceptualized and realized several qualitative interview studies previously |
| Relationship with participants | |
| 1. Relationship established | The interviewees were part of the medical education program and therefore known to some of the authors as they are responsible for the training (TB, PB, AS). The authors who collected the data or analysed them (JG, NB, SG) had no prior existing relationship with the interviewees. |
| 1. Participant knowledge of the interviewer | Interviewees were informed about the project, interviewer’s educational background and occupational status in advance. Participants had the chance to request further information regarding the provided information. |
| 1. Interviewer Characteristics | The interviewers have a research interest in health services research and sociology, especially in the area of professionalization and narrative perspectives on medical students |
| Domain 2: Study design | |
| Theoretical Framework | |
| 1. Methodological orientation and theory | Reported in the methods section “Analysis”: Transcripts of the semi-structured topic guide interviews were analysed using a Grounded Theory Approach in combination with the methodological approach of spatial methods. |
| Participant selection | |
| 1. Sampling | Reported in the methods section. |
| 1. Method of approach | Reported in the methods section. |
| 1. Sample size | Reported in the methods section. |
| 1. Non-participation | Reported in the methods section. |
| Setting | |
| 1. Setting of data collection | Reported in the methods section “Data collection”:  Interviews: professionals’ workplaces or at home.  Additional information: Interviewees attended the call either at their workplaces or at home. |
| 1. Presence of non-participants | No one else was present besides the participants and the interviewer. |
| 1. Description of Sample | Reported in the methods section. |
| Data Collection | |
| 1. Interview Guideline | The development of the interview guide is briefly described in the methods section “Data collection”. |
| 1. Repeat interviews | No repeat interview was necessary. |
| 1. Audio/Visual recording | Reported in the methods section “Data collection: Interviews were audio-recorded and transcribed verbatim, including pseudonymization. |
| 1. Field notes | No field notes were written. |
| 1. Duration | Not reported: Interview duration ranged between # and # minutes. |
| 1. Data saturation | Reported in the methods section “Data collection”. |
| 1. Transcripts returned | Transcripts could not be returned to participants due to pseudonymization, which was demanded by our data protection officer. However, interviewers continuously mirrored and confirmed descriptions during the interview to guarantee correct understanding. |
| Domain 3: Analysis and findings | |
| Data analysis | |
| 1. Number of data coders | Reported in the methods section “Analysis”:  Indexing all interviews and analysing: JG, SG  Summarizing and charting of the indexed data: JG, NB, SG |
| 1. Description of the coding tree | Reported in the methods section “Analysis”. |
| 1. Derivation of themes | Reported in the methods section “Analysis”. |
| 1. Software | Reported in the methods section “Analysis”. |
| 1. Participant checking | Not reported. |
| Reporting | |
| 1. Quotations presented | Quotations from different participants are presented to illustrate the findings, and a number identifies each quotation. |
| 1. Data and findings consistent | Yes |
| 1. Clarity of major themes | The ideas and experiences of rurality emerged as an important aspect in the context of the broader research interest in longitudinal perspectives on the program as well as the professionalization of medical students. |
| 1. Clarity of minor themes | As far as the word count permits, we discuss minor themes, too. |
