# Supplementary material for: Primary care in rural areas: a qualitative study on medical students’ images and experiences of working in rural areas in southern Germany
Source: BMC Prim Care. 2024 Dec 16;25:416. doi: 10.1186/s12875-024-02677-x (PMC11648291; doi:10.1186/s12875-024-02677-x)
Supplement: Supplementary file 2 — Supplementary Material 2 [file 12875_2024_2677_MOESM2_ESM.pdf]

Key Topics	Questions
**0) Introduction**	• Can you please briefly introduce yourself? (Origin: rural area? Parents: doctors?)<br>• How did you come to study medicine?<br>• What are you currently doing? What stage of medical education are you in?
**1) Evaluation of BeLA**	• Why did you choose to participate in the BeLA programme?<br>• What did your everyday life looked like?<br>• Did you have any doubts about going to the country? Were there any doubts about taking part in the programme? Which ones?<br>• What did you particularly like? What didn't work out so well?<br>• What should definitely be retained? What should be changed?<br>• Do you feel well prepared for medical practice?
**2) Biographical decision**	• How did you find out about the Bela programme?<br>• What convinced you to participate?<br>• Did you have any doubts about taking part in the programme? Which ones?<br>• Generally speaking, is it difficult to make biographical decisions so early on in your education?

	• What is problematic about "commitments" in general?<br>• Do you have the feeling that it is difficult to reconcile work and family life?<br>• How did you come to general medicine in the first place?<br>• What are your expectations for the time after BeLA?<br>• Are you planning to specialise in general medicine?<br>• Why? Why not?<br>• Can you imagine working as a rural practitioner later on? In your own medical practice?<br>• Can you imagine working and living in the rural region?<br>• Have you already made a decision?<br>• Why not? Why are you unsure? What are the main factors?
**3) Individual perception of General Medicine**	• What characterises a typical general practitioner? How would you characterise them? What characterises a rural practitioner?<br>• How would you describe the image of general practice? In general in society & among students?<br>• Has your image of general practice changed during your participation in the Bela Programme?

**4) Conclusion**	Is there a particular moment that has changed your perception?<br><br>• Who does the programme reach? Who does it not?

Key Topics	Questions
**0) Introduction (if necessary)**	• Can you please briefly introduce yourself? (Origin: rural area? Parents: doctors?)<br>• How did you come to study medicine?
**1) Evaluation of BeLA**	• What are you doing right now?<br>• Why did you choose to participate in the BeLA programme?<br>• What did your everyday life looked like?<br>• Did you have any doubts about going to the country? Were there any doubts about taking part in the programme? Which ones?<br>• What did you particularly like? What didn't work out so well?<br>• What should definitely be retained? What should be changed?
**2) Biographical Decision**	• Has the practical experience of the BeLA programme changed your view of General Practice?<br>• What are your expectations for the time after BeLA?<br>• Are you planning to specialise in general medicine?<br>• Why? Why not?<br>• Can you imagine working as a rural practitioner later on? In your own medical practice?<br>• Can you imagine working and living in the rural region?

	• Have you already made a decision?<br>• Why not? Why are you unsure? What are the main factors?
**3) Uncertainty in medical practice**	• Have you experienced situations in practice in which you experienced uncertainty? In diagnostic/therapeutic decisions.<br>• Did you feel that you were well prepared for these situations?<br>• How did you deal with these situations?<br>• Can you remember a specific situation/example?
**4) Individual perception of General Medicine**	• What characterises a typical general practitioner? How would you characterise them? What characterises a rural practitioner?<br>• How would you describe the image of general practice? In general in society & among students?<br>• Has your image of general practice changed during your participation in the Bela Programme?<br>• Is there a particular moment that has changed your perception?

**5) Conclusion**	• Who does the programme reach? Who does it not?

Key Topics	Questions
**1) Evaluation of BeLA**	• What are you doing right now?<br>• Why did you choose to participate in the BeLA programme?<br>• What did your everyday life looked like?<br>• Did you have any doubts about going to the country? Were there any doubts about taking part in the programme? Which ones?<br>• What did you particularly like? What didn't work out so well?<br>• What should definitely be retained? What should be changed?
**2) Biographical Decision**	• Has the practical experience of the BeLA programme changed your view of General Practice?<br>• What are your expectations for the time after BeLA?<br>• Are you planning to specialise in general medicine?<br>• Why? Why not?<br>• Can you imagine working as a rural practitioner later on? In your own medical practice?<br>• Can you imagine working and living in the rural region?<br>• Have you already made a decision?<br>• Why not? Why are you unsure? What are the main factors?

**3) Uncertainty in medical practice**	• Have you experienced situations in practice in which you experienced uncertainty? In diagnostic/therapeutic decisions.<br>• Did you feel that you were well prepared for these situations?<br>• How did you deal with these situations?<br>• Can you remember a specific situation/example?
**4) Individual perception of General Medicine**	• What characterises a typical general practitioner? How would you characterise them? What characterises a rural practitioner?<br>• How would you describe the image of general practice? In general in society & among students?<br>• Has your image of general practice changed during your participation in the Bela Programme?<br>• Is there a particular moment that has changed your perception?
